# Small-livestock farmers’ perceived effectiveness of predation control methods and the correlates of reported illegal poison use in the South African Karoo

**DOI:** 10.1007/s13280-023-01892-7

**Published:** 2023-06-30

**Authors:** Marine Drouilly, Nicoli Nattrass, M. Justin O’Riain

**Affiliations:** 1 Institute for Communities and Wildlife in Africa, H.W. Pearson Building, University of Cape Town, University Avenue North, Rondebosch, Cape Town, 7701 South Africa; 2 Panthera, 8 W 40th Street, 18th Floor, New York, NY 10018 USA; 3 Centre for Social Science Research, Robert Leslie Social Science Building, University of Cape Town, 12 University Avenue South, Rondebosch, Cape Town, 7701 South Africa

**Keywords:** Caracal, Human-wildlife conflict, Jackal, Livestock predation, Poison, Predator management

## Abstract

The use of poison against predators is pervasive and negatively impacts biodiversity and ecosystem health globally. Little is known about the correlates of poison use as a lethal control method on small-livestock farmland. We used a mixed-methods approach to investigate commercial farmers’ experience with and perceived effectiveness of predation control methods, reported poison use and its correlates in the Central Karoo. Farmers perceived lethal methods to be cheaper and more effective than non-lethal methods in protecting their livestock from predation. They reported more experience with lethal methods, and over half reported having used poison. This is higher than other estimates in southern Africa and consistent with other survey-based evidence from the Karoo. Reported poison use was positively related to perceived efficacy, declining on-farm employment and perceived threats of predators. It was negatively related to terrain ruggedness. Our findings provide an understanding of the context and motivations shaping this illegal behavior.

**Supplementary Information:**

The online version contains supplementary material available at 10.1007/s13280-023-01892-7.

## Introduction

Lethal control of predators is a widespread and longstanding human practice (Reynolds and Tapper 1996). Although diverse non-lethal methods have been developed and promoted (e.g., Tobajas et al. 2019; Naha et al. 2020), lethal control remains pervasive, mostly because of its perceived effectiveness in managing “problem” or “damage-causing” animals (Scasta et al. 2017). Livestock predation is the major cause of negative interactions between people and carnivores worldwide (Torres et al. 2018), due to its socio-economic (Gallardo et al. 2020), psychological (Chowdhury and Jadhav 2012) and health impacts (Barua et al. 2013) on individual farmers and rural communities. Predators are lethally removed from ecosystems in an effort to extirpate or to keep their populations within desired limits and with the goal of maintaining livestock-related activities (Woodroffe et al. 2005). This includes lions (*Panthera leo*), leopards (*Panthera pardus*) and spotted hyenas (*Crocuta Crocuta*) in Africa (Kissui 2008); dholes (*Cuon alpinus*) in Asia (Lyngdoh et al. 2014); jaguars (*Panthera onca*) in South America (Jędrzejewski et al. 2017); dingoes (*Canis lupus dingo*) in Australia (Edwards et al. 2021); and wolves (*Canis lupus*), foxes (red foxes *Vulpes vulpes*) and bears (brown bears *Ursus arctos*) in North America (Scasta et al. 2017) and Europe (Fernández-Gil et al. 2016).

The most common methods to control predators are shooting, trapping and poisoning (Woodroffe et al. 2005). The illegal use of poison baits to control species that predate on livestock or game, or that cause damage to food stores or crops, occurs in all landscapes, from agroecosystems (Márquez et al. 2013) to urban areas (Vogler et al. 2015), and even within protected areas (Ntemiri et al. 2018). Illegal poisoning often targets raptors and mammals that include scavenging as means to obtain food, and can have devastating effects on non-target wildlife through secondary poisoning (Mateo-Tomás et al. 2012). It also presents an important risk to many threatened species (Santangeli et al. 2016) through direct and delayed chronic effects (Grue et al. 1997), to the environment through cascading effects, and to people (UNEP 2016).

Livestock predation in South Africa has been estimated to cost USD 164 million per annum (Turpie and Babatopie 2018). Predator control by small-livestock farmers has a long history, dating back to 1652 (Beinart 1998; Nattrass et al. 2020b), with programs mostly targeting black-backed jackals (*Canis mesomelas*, hereafter jackal). The passing of the Fencing Act and the spread of enclosures in 1883 to keep livestock within selected areas and predators out encouraged farmers to increase their use of poison within fenced areas, often with devastating effects on the populations of various animals, including non-target species (van Sittert 2016). In 1973, the Hazardous Substances Act restricted the use of sodium cyanide (1080), with the result that small-livestock farmers in the arid Central Karoo began experimenting with a variety of readily available agrochemicals as poisoning agents (Nattrass and Conradie 2015). The decline in government support for both fencing and predator management from the 1980s resulted in the recolonization of the Karoo by jackals in a context where small-livestock farmers had fewer resources to manage them (Drouilly et al. 2018b; Nattrass et al 2020b). The result was rising predation rates, with a reported mean of livestock losses of 89% in the South African Karoo in 2014 (Nattrass and Conradie 2018).

Few studies have investigated the use and efficacy of non-lethal and lethal methods, including poison, to limit predation (Kerley et al. 2018). Evidence from the Karoo suggests that killing predators is associated with increased livestock losses the following year (Nattrass et al. 2020a, b), though most farmers continue to employ lethal control in retaliation for livestock losses (Nattrass and Conradie 2018). Research conducted in Namibia (Santangeli et al. 2016) estimated that 20% of commercial farmers used poison to control predators—and that rose to 50% in areas dominated by small-livestock farming. Research in north-eastern South Africa (St John et al. 2012) and in areas to within 100 km of vulture range (Brink et al. 2021) found that about a fifth of farmers probably used poison, with the highest prevalence found in arid areas used for small-livestock farming. A longitudinal study of the economics of sheep farming in the Karoo found that reported poison use rose from 22 to 50% between 2012 and 2014 (Nattrass and Conradie 2018).

This article focusses on sheep farmers in South Africa’s Central Karoo District Municipality, a long-standing sheep farming area that experiences less than 150 mm of rain per year (for more details, see Drouilly et al. 2021). Jackal and caracal (*Caracal caracal*) are perceived as most responsible for livestock losses in this area and during 2014–2015, we recorded 706 jackals and 383 caracals killed pre-emptively or in retaliation for livestock losses. This was inevitably an underestimate and fails to include most deaths through poison. Some farmers also list chacma baboons (*Papio ursinus*; hereafter, baboons) and Cape fox (*Vulpes chama*) as a threat to their livestock, although to a lesser extent than jackal and caracal (Drouilly et al. 2021). The small size of the Cape fox also limits predation to lambs < 3 months old (Kok and Nel 2004).

We probe the reasons given by farmers and the context for lethal management of predators and describe how farmers perceived the effectiveness of different methods. As most farmers equated killing predators with protecting livestock, perceived effectiveness was understood in the same way—as simultaneously effective in killing predators and protecting their livelihoods.

We draw on interviews and ethnographic insights to explore potential economic and attitudinal drivers of reported poison use. Given that cases of wildlife poisoning on private land are almost impossible to detect and are seldom reported, our research provides novel insight on some correlates of the illegal reported use of poison, which is the first step in considering potential approaches for mitigating its use and the damage it causes to the environment.

## Materials and methods

We used a mixed-methods approach in which our questionnaire design and empirical analysis was informed by ethnographic field observations of farmers in the Central Karoo. Before conducting the semi-structured interviews between July 2014 and March 2015, the interviewer (first author) lived for 22 months from September 2012 to June 2014 on the farm of one of the respondents and the other authors visited the area regularly and engaged with farmers. The first author participated in the daily life and duties of farmers (e.g., assisting with sheep management, following along during night hunts and attending agricultural meetings). Such ethnographic immersion allowed her to obtain a degree of “insider knowledge” (Bucerius 2013) and to understand the social context. Direct observation of various behaviors (including poison use) allowed for the triangulation of findings and increased data reliability. In addition, farmers regularly discussed their experience with predator control, including poison. Our participation in such discussions during formal and informal meetings with farmers positioned us socially as people who understood that poison use was widespread, and thus encouraged farmers to be open with us on the issue (Puri 2010). We used some of the rich qualitative data captured during the ethnographic process to discuss the quantitative results obtained during the interviews.

Interview respondents were approached through agricultural organizations and word-of-mouth. We were able to reach 77 small-livestock farmers, i.e., at least 64% of farms recorded in the most recent agricultural census of the Central Karoo District Municipality (i.e., 2014/2015; Western Cape Department of Agriculture 2017). To the extent that there was selection bias, it was towards farmers living in the area and who participated in local farming networks. None of the farmers we approached declined to participate and all interviews were conducted on farms, and in person. The questionnaire instrument was developed after extensive informal and open-ended discussions and was piloted before finalizing. Interviews were conducted by the first author in English, with Afrikaans translations available if needed. Each interview lasted about one hour. Ethical approval was received from the University of Cape Town (UCT/COM/012/2012) (also see Appendices S1 and S2 for more details on the interview process and S3 for the set of questions relevant to this manuscript).

### Farmers’ experience and perceived effectiveness of predation control methods

We asked farmers a series of questions about demographic and socio-economic characteristics, whether they had livestock losses on their farms and if so, what they thought was the main cause. We asked whether they attempted to limit livestock predation and if so, what methods they used and the perceived effectiveness of each method using a five-point rating scale from 1 (does not work at all) to 5 (works very well) for jackal and caracal separately. We transformed the five-point rating scale into a three-point rating scale (i.e., “works well”, “works a little” and “does not work”) because we had low frequencies for some of the categories. We also asked farmers how they usually respond to fresh signs (e.g., spoor, scat) of jackal and caracal on their farms. We assessed whether there was a difference in the perceived effectiveness and cost of lethal versus non-lethal methods by comparing the mean score given by farmers to each type of methods with a Student’s *t* test. We used a *χ*^2^ test to assess the null hypothesis that there is no relationship between type of method used (i.e., lethal versus non-lethal) and its relative cost.

### Farmers’ reported use of illegal poisons

We asked farmers whether they believed poison was effective against predators (possible answers: “yes”, “no”, “unsure”), whether they had used poison on their farms in the last 5 years (“yes”, “no”, “prefer not to answer”) and if so, how often (“regularly” (i.e., at least once a month), “as a last resort” (i.e., when farmers considered that no other methods had been effective in limiting livestock losses), “not using poison”, “never”, “prefer not to answer”). We recorded the experience of farmers with different types of poisons.

Sensitive questions about poison use were asked towards the end of each interview to ensure that farmers were as comfortable as possible with the interview process.

Direct questions about poison use pose a particular challenge as they can result in two forms of survey bias: fear of legal sanction (arising out of concern that the information provided might not remain confidential); and social desirability bias (arising out of the interviewee wanting to avoid embarrassment and to project a favorable image to the interviewer, so they won’t be judged negatively by them) (Tourangeau and Yan 2007). Poison use is illegal in South Africa and for this reason, some have used indirect methods such as the randomized response technique (St John et al. 2012) or the list experiment (Brink et al. 2021) to estimate it. We assessed at the time that farmers would be suspicious of indirect questioning strategies (and that it could cause skepticism and wariness about what the researcher was trying to do, Tourangeau and Yan 2007) and by then were confident that farmers were prepared to talk to us about poison use. Both studies using indirect questioning methods found that perceptions of peer behavior were important determinants of poison use (St John et al. 2012; Brink et al. 2021). Our ethnographic immersion in the study site both allowed us to uncover what was clearly a widespread social practice and to communicate to interviewees that we understood this and would not be judgmental. This, we argue, would reduce social desirability bias. During informal discussions, we also recorded information showing that the farming community had come to trust us (e.g., “I will tell you all the methods I use, even poison, because we want to do research. We need to find solutions for the farmers.” Farmer #02), and thus we hoped that they would be less fearful of us potentially reporting them to the authorities (that is, violating the promised confidentiality of the interviews). This would reduce bias arising out of fear of legal sanction. That it was also very difficult for the authorities to police illegal poison use on these extensive rangelands (we knew of no one in the study area who had been warned or prosecuted) likely also reduced this source of potential bias.

To explore potential correlates of poison use quantitatively, we used a binary logistic regression where 1 denotes a farmer who reported using poison on his farm(s) either regularly or as a last resort, and 0 denotes a farmer who reportedly never has (“never”) or does not currently (“not using poison”) use poison. We selected a set of 10 a-priori variables as likely covariates of reported poison use based on the above literature review, ethnographic field observations and our discussions with farmers, and formulated predictions (Table 1; more information about the model selection process can be found in Appendix S4).

**Table 1:** Description and expected signs of the variables hypothesized to influence reported poison use on small-livestock extensive farms in the South African Karoo. Variables were included as predictors within the binary logistic regressions. Mean and range values are provided when appropriate. Except if stated otherwise, all variables were extracted from the face-to-face semi-structured interviews with farmers

Variables of expected importance	Type	Mean [range]	Description	Expected sign of influence on reported poison use and justification
Fall in employment on the respondent’s farm(s)	Discrete	[− 9 to 8]	Difference between the number of farm workers on the farm at the time of the interview and when the farmer started farming	(+): A lower number of laborers since the respondent had started farming is predicted to lower the effort spent on patrolling, maintaining border fences, actively monitoring/tracking/trapping predators and on caring for sheep/lambs. A fall in employment also proxies for economic hardship
Jackal, caracal and baboons in the top three predators	Boolean	Yes/no	Whether jackal, caracal and baboons were ranked as the three main predators causing livestock losses on the respondent’s farm(s)	(+): The presence of those three predators is linked to higher reported overall losses on farms (Drouilly et al. 2018b). In Namibia, farmers who suffered high livestock losses were most likely to admit to using poison (Santangeli et al. 2016). These predators are also very adaptable to human persecution and to various control methods (Drouilly et al. 2018b)
Cape fox in the top five predators	Boolean	Yes/no	Whether Cape fox was ranked as one of the five main predators causing livestock losses on the respondent’s farm(s). Drouilly et al. (2021) found that Cape fox was most often ranked fifth by farmers in terms of predation in the area	(+): The respondent would be less concerned about killing Cape fox as a non-target species if it is also believed to cause losses
Farmer considers poison effective against predation	Boolean	Yes/no	Whether the respondent believes poison is effective at killing predators	(+): The respondent would be more inclined to use illegal poisoning if he perceives it as being effective
Believing in the dominant jackal pair narrative	Boolean	Yes/no	Whether the respondent believes that killing a territorial jackal pair (i.e., disrupting jackal social structure) will result in an increase in jackal numbers (due to dispersing individuals filling the vacuum created), and hence higher livestock losses (reported in Nattrass and Conradie 2015 in the context of the study area)	(−): Killing of territorial jackal pairs has been linked to disruption in social structure and to immigration of new individuals to occupy the vacant territories (Minnie et al. 2018; Tensen et al. 2018)
Age	Continuous	50.6 [25–76]	Respondent age	(±): Mateo-Tomás et al. (2012) showed that there was no significant influence of age on the illegal use of poison in Spain. However, poison use might be more prevalent among older farmers who started farming when this practice was widespread and more acceptable (Brink et al. 2021). Older farmers might also be less inclined to go through the physicality and discomfort of night hunting and therefore may more easily resort to poison use. On the contrary, younger men may have more motivation to hunt than to use poison, because hunting is a form of sport and an occasion to socialize (pers. obs.). However, evidence from the Karoo suggests that age is negatively related to poison use (Nattrass and Conradie 2018)
Number of years of education	Discrete	[10–15]	Respondent’s number of completed years of formal education	(−): Farmers with more years of education might be more aware of the unintended ecological impacts of this behavior
Percentage of lambs lost	Continuous	Probability units 0.294 [0.01–0.81]	Perceived percentage of lambs lost attributed to predators on the respondent’s farm(s)	(+): Farmers with higher livestock losses were shown to be more inclined to use poison (Santangeli et al. 2016; Brink et al. 2021; Didarali et al. 2022)
Relative Terrain Ruggedness Index (TRI)	Continuous	Index 0.36 [0.14–1.00]	Relative mean of the absolute values of the differences between a central pixel and its neighbors (sensu Riley et al. 1999; Wilson et al. 2007). The most rugged farm has a value of 1. All the other farms are relative to the most rugged. TRI was calculated using the Digital Elevation Model (DEM) layer of the Western Cape (30 m precision) in QGIS 2.18.2	(±): In northern Spain, the use of illegal poison increased in mountainous areas where wolves frequently predated on livestock because rugged areas are difficult to patrol, with reduced surveillance of livestock, notably by shepherds and dogs (Mateo-Tomás et al. 2012). It is possible that similar dynamics would be evident on the Karoo farms, although it is possible that most farmers deploy poison where it is easy to do so, i.e., on flat plains where there is road access, in the hope that predators, which move large distances, will pick up the baits at some point
Total farms size	Continuous	Kilometers squared 83.74 [5.70–260]	The cumulative area of all the farms of the respondent (*i.e.*, main farm and additional portions). Farm size was extracted from the regional cadastral map (Farm Portions, Department of Rural Development and Land Reform, Chief Surveyor-General Office, Western Cape, 2013) in QGIS 2.18.2 (QGIS Development Team, 2017)	(±): Livestock scattered across large areas can be more difficult to protect. Larger farms also require more effort to patrol, with more fences to check and maintain, and hence poisoning might be used more than on smaller farms, as was shown in Namibia (Santangeli et al. 2016) and northern South Africa (St John et al. 2012). On the other hand, having a larger farm may mean that the farmer is financially more secure and may thus be able to absorb more losses before resorting to the use of an illegal method

## Results

### Farmers characteristics and farming context

All respondents were men, which was to be expected given that patriarchy is a cultural norm in the study area, with men typically assuming responsibility for farm operations and predator control. Respondents had an average of 25 years of farming experience in the Karoo (SD = 13.2, range 1–56), 85.7% had attained a school leaving certificate and 58.4% had some level of tertiary education (18.2% had a university degree). About a third (39%) held a National Diploma in Agriculture from the Grootfontein Agricultural Development Institute (one of the oldest agricultural colleges in South Africa). Small-livestock farming in the area is often economically marginal and rising minimum wages has put pressure on employment (Nattrass and Conradie 2018). In our sample, the number of full-time workers per farm had decreased from a median of three when the farmers had commenced farming to two by 2014 (Table 1). All the respondents were Christians. Small-livestock farming (dominated by Dorper sheep) was the main economic activity on the farms. Respondents owned an average of 1205 ewes (SD = 1210.6, range 0–6000) and the average stocking rate was 144 ewes/1000 hectares. Of the respondents, 98.7% claimed to have lost livestock in the last year and predators were perceived by 81.2% of the respondents as the main cause of livestock losses, followed by theft (Appendix S5).

### Farmers’ experience and perceived effectiveness of predation control methods

**Fig. 1:**
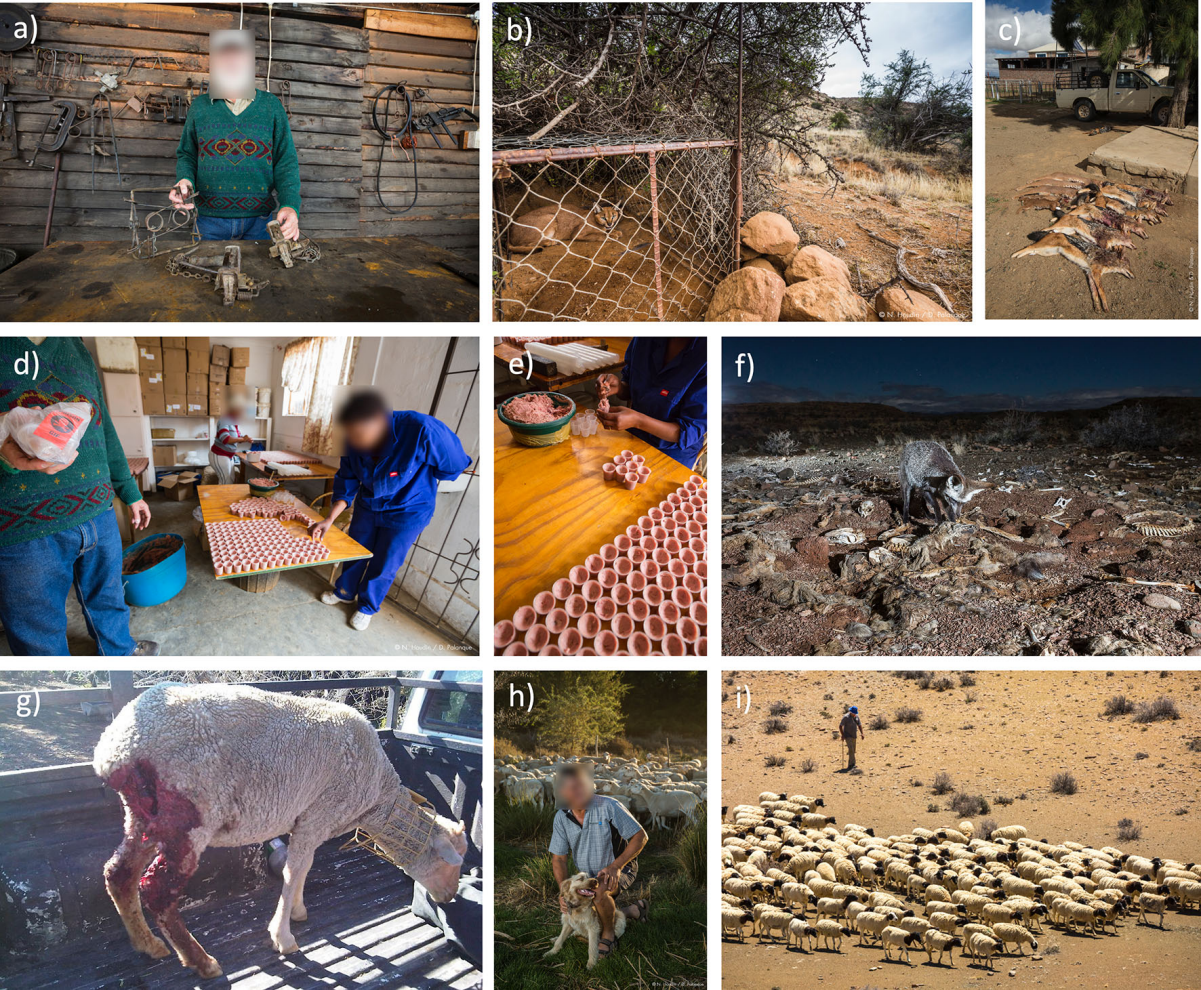
Photographs taken in the South African Central Karoo in 2016 showing lethal and non-lethal methods used by farmers to control predators: **a** lethal gin and conibear traps; **b** caracal caught in a walk-in cage trap on a farm; **c** jackals and caracals shot at night with the “call and shoot” technique typically effected from a vehicle equipped with powerful lights; **d** and **e** poison (“gif” in Afrikaans) sold in the form of “vetpil”, a mixture of meat, animal fat and sodium cyanide that could be easily obtained from the legal supplies of a local licensed dealer in the Karoo; **f** “predator cemetery” where farmers throw away the carcasses of predators they kill during lethal control operations, including poisoned animals. Here, a non-target bat-eared fox (*Otocyon megalotis*) scavenges on the remains and risks secondary poisoning; **g** an injured shearling that was bitten on the rump, highlighting the fact that its protective collar was ineffective against black-backed jackal; **h** one of the sheep farmers with his livestock-guarding dog; **i** herding sheep can be an effective non-lethal practice, but it has almost disappeared from the area. © N. Houdin & D. Palanque, except **g** © Karoo Predator Project

**Fig. 2:**
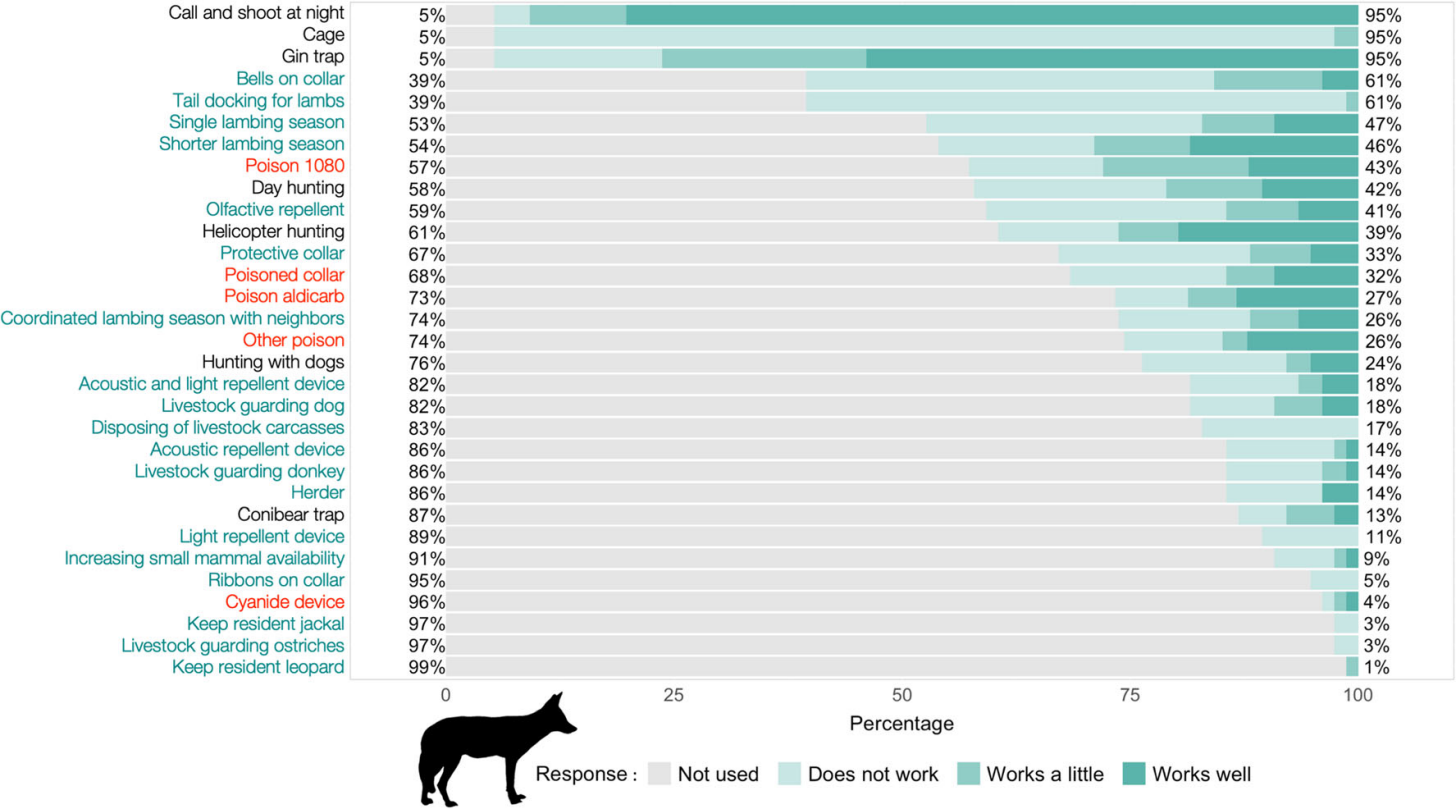
Percentage of farmers who have experience (shades of green) or not (grey) with a range of control methods against jackal predation on their farms. When the farmers had experience with the method, its perceived effectiveness was recorded at three levels: “does not work” (palest shade of green), “works a little”, “works well” (darkest shade of green). On the *y*-axis, lethal methods are in black font, poisons are in red and non-lethal methods are in green. The percentages on the right *y*-axis represent the approximate percentages of farmers with experience of the different methods

**Fig. 3:**
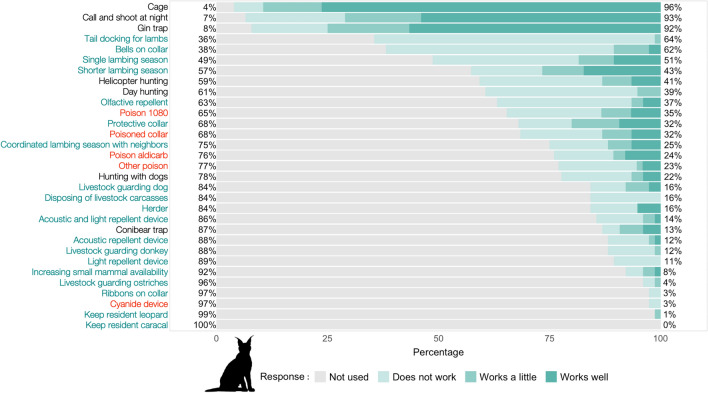
Percentage of farmers who have experience (shades of green) or not (grey) with a range of control methods against caracal predation on their farms. When the farmers had experience with the method, its perceived effectiveness was recorded at three levels: “does not work” (palest shade of green), “works a little”, “works well” (darkest shade of green). On the *y*-axis, lethal methods are in black font, poisons are in red and non-lethal methods are in green. The percentages on the right *y*-axis represent the approximate percentages of farmers with experience of the different methods

**Fig. 4:**
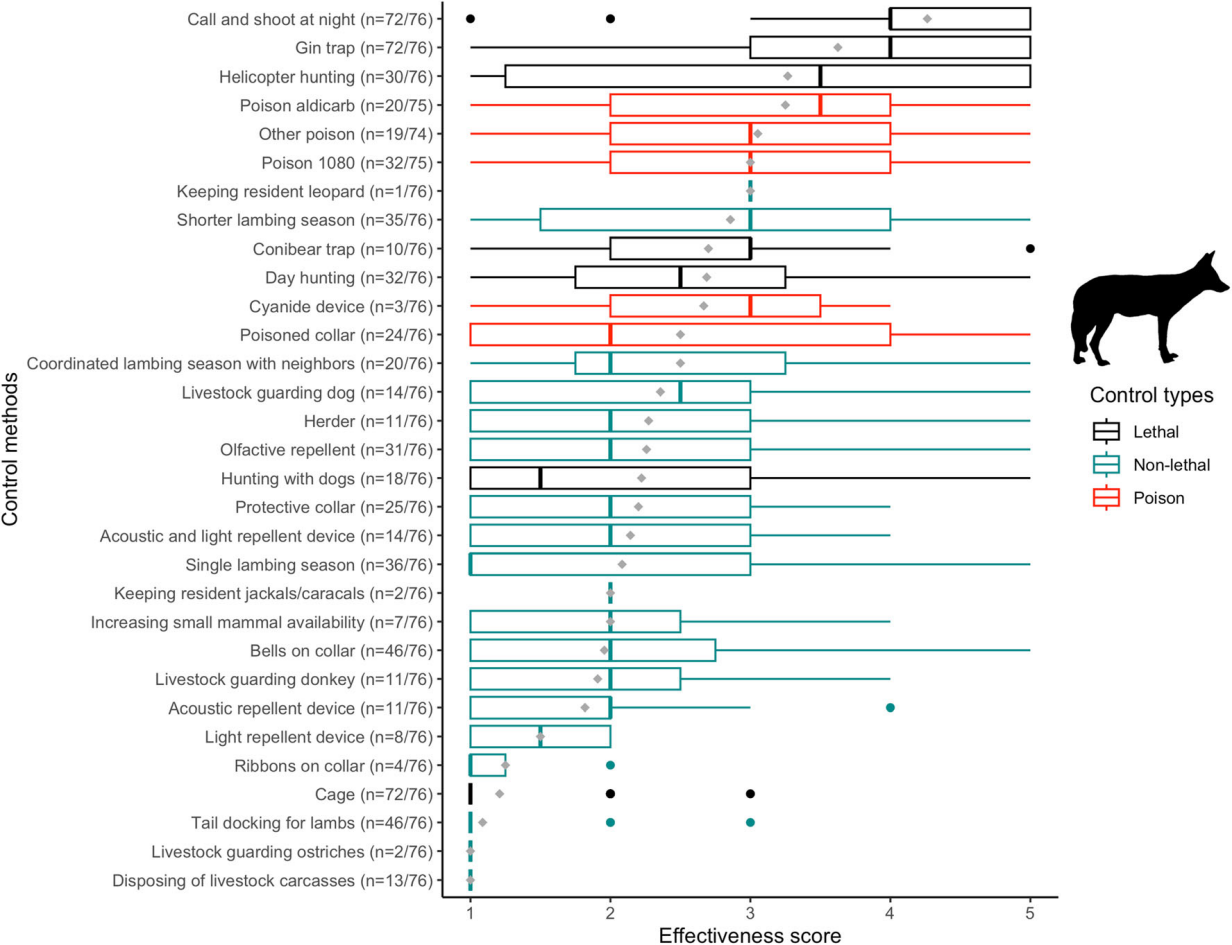
Boxplot of farmers’ perceived effectiveness scores for a range of control methods against black-backed jackal (*Canis mesomelas*) predation in the Karoo. Lethal methods are in black, poisons are in red and non-lethal methods are in green. The grey diamond shapes represent the mean scores for each method and the dots are outliers

Almost all (97.4%) farmers reported using lethal methods to reduce predation by jackal and caracal (Figs. 2, 3), the most common being: (1) call and shoot at night—which involves playbacks of jackal conspecific challenge calls, or prey-in-distress calls; (2) gin traps, which are unpadded with offset jaws that are triggered when the animal steps on a trigger plate; and 3) walk-in cage traps (Figs. 1, 2, 3). For jackals, only calling and shooting at night was reported as being very effective (here, “effective” refers to the methods ability to capture predators, and farmers make the assumption that if a method is better at capturing predators, then it deters predation; mean effectiveness score: 4.3/5), whereas gin traps were reported as relatively effective (3.6/5) and cage traps as ineffective (1.2/5), mostly because only the young inexperienced jackals were reported to be trapped in cages (Fig. 4). Cage trapping was considered the most effective method against caracals (mean effectiveness score: 4.1/5), whereas gin traps (3.7/5) and calling and shooting at night (3.4/5) were reported as being relatively effective on average.

**Fig. 5:**
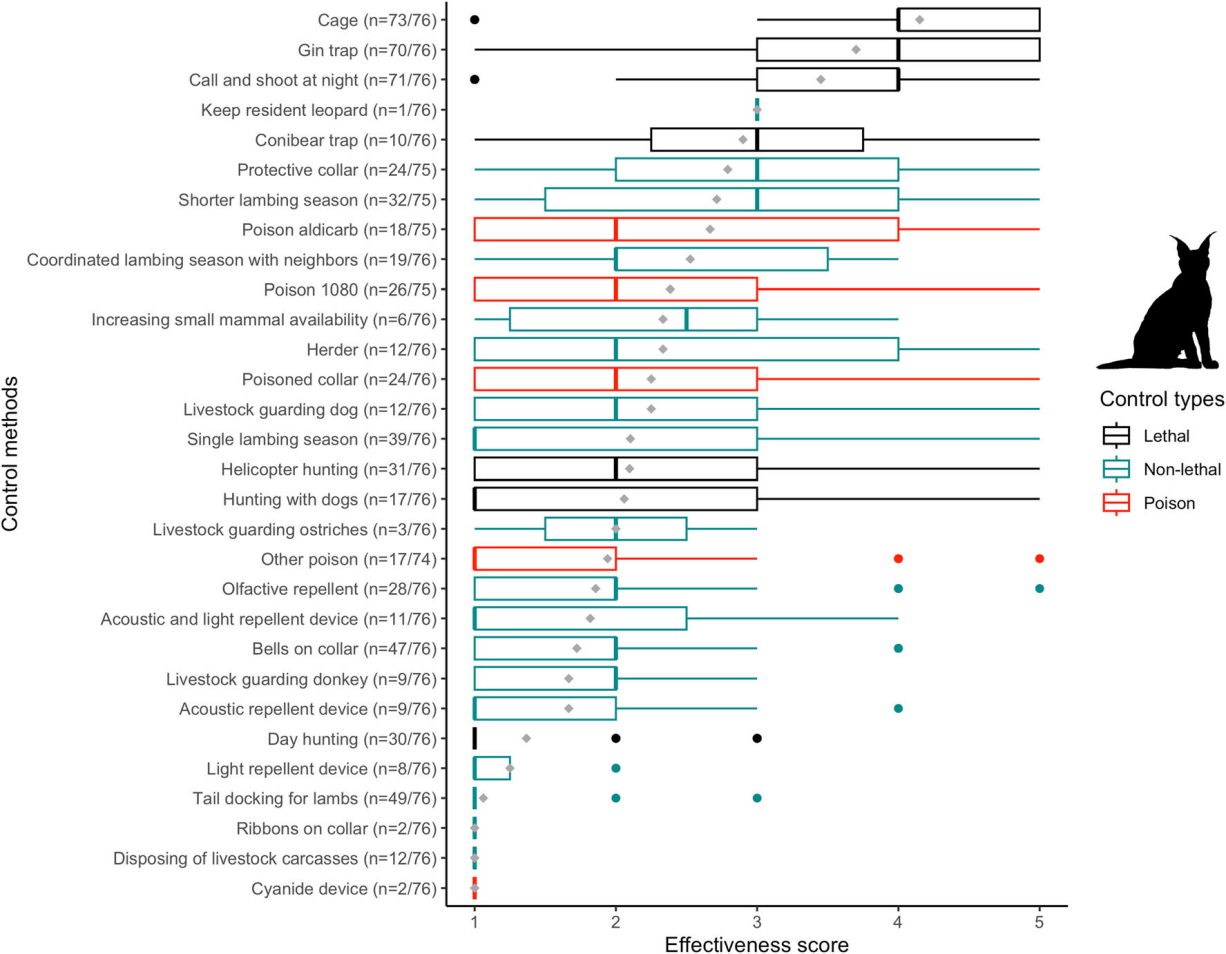
Boxplot of farmers’ perceived effectiveness scores for a range of control methods against caracal (*Caracal caracal*) predation in the Karoo. Lethal methods are in black, poisons are in red and non-lethal methods are in green. The grey diamond shapes represent the mean scores for each method and the dots are outliers

Few farmers had experience with non-lethal methods (Figs. 2, 3) other than jackal-proof fencing, which was used by all farmers (although with different degrees of maintenance). Attempts were made to adapt husbandry to reduce livestock losses to predators, including having a single and/or shorter lambing season (< 50% of respondents) and coordinating lambing season with that of the neighbors’ (ca. 25% of respondents) (Figs. 2, 3). Equipping lambs with protective collars (reported as being inexpensive but labor intensive as they require the adjustment of the straps of the collars as the lambs grow) was used by 32% of the farmers with some success against caracals, but the effectiveness score ranged from 1 to 5, showing it was not a one-size-fits-all solution (Figs. 1, 5).

The mean perceived effectiveness score of lethal control methods was significantly higher than that of non-lethal methods for jackals (*t* = 4.04, df = 28, *p* < 0.001) but not for caracals (*t* = 1.94, df = 28, *p* = 0.06). Most farmers (59.7%) thought that using non-lethal methods was more expensive than lethal methods (*χ*^2^ = 15.00, df = 1, *p* < 0.001), but 11.7% said they did not know.

### Prevalence and correlates of reported poison use

Farmers were open to discussing predator control methods with us and most reported using poison (e.g., “I’m not going to lie to you, I use poison and you saw the jackal cemetery on my farm, but what are the alternatives that work?” Farmer #19; “I am using poison as a last resort. I know it is bad for my steenbokkie [i.e., affectionate Afrikaans term for steenbok (*Raphicerus campestris*)] and hares and the small wildlife, but the jackals will eat all my sheep” Farmer #01). Only one farmer declined to answer our questions about poison use. Of the remaining 76 farmers, half reported using poison, either regularly (17%) or as a last resort (33%) for dealing with the most elusive and difficult-to-catch individuals. Of the farmers who used poison regularly, all believed that it was an effective means of reducing predation (Table 2). More than a tenth of the respondents declared that they would immediately deploy poison in response to fresh signs of jackals or caracals on their farms. Approximately a quarter of the respondents reported that they had used, possessed or traded in the illegal agricultural pesticide known as “two-step” (i.e., aldicarb, which is a carbamate insecticide and the active substance in the pesticide Temik®), to reduce predator numbers (Figs. 2, 3), with a mean perceived effectiveness of 3.2 for jackal and 2.7 for caracal (Figs. 4, 5). More than a third of farmers reported having used sodium fluoroacetate (commonly called “1080”) against both predators (Figs. 2, 3), with a perceived effectiveness of 3.0 for jackal and 2.4 for caracal. About a quarter of farmers reported that they had also used “other” poisons considered to be effective against jackals (mean score of 3.0; Fig. 4) without always mentioning which one(s) (Figs. 2, 3). Poisons were usually laced in baits taking the form of “vetpil”, a mixture of meat, animal fat and sodium cyanide hung from branches or hidden in bushes close to signs of predators or kill sites (Fig. 1).

**Table 2:** Reported frequency of use of various types of poisons by small-livestock farmers (*n* = 76) in the South African Karoo, and whether or not respondents think the poison is effective against predators. “Regularly” refers to at least once a month. “Last resort” applies when no other methods (such as calling and shooting at night) has been effective in limiting livestock losses on the respondent’s farm

		Experience with different types of poisons			Respondents think poison is effective against predators		
		1080	Aldicarb	Other	Yes	No	Unsure
Use poison regularly	17% (13/76)	75% (9/12)	42% (5/12)	58% (7/12)	100% (13/13)	0% (0/13)	0% (0/13)
Use poison as a last resort	33% (25/76)	56% (14/25)	44% (11/25)	32% (8/25)	84% (21/25)	12% (3/25)	4% 1/25)
Do not use poison	50% (38/76)	22% (8/37)	8% (3/37)	14% (5/37)	42% (16/38)	47% (18/38)	11% (4/38)
Total	100% (76/76)	43% (32/75)	27% (20/75)	27% (20/74)	66% (50/76)	28% (21/76)	6.6% (5/76)

**Fig. 6:**
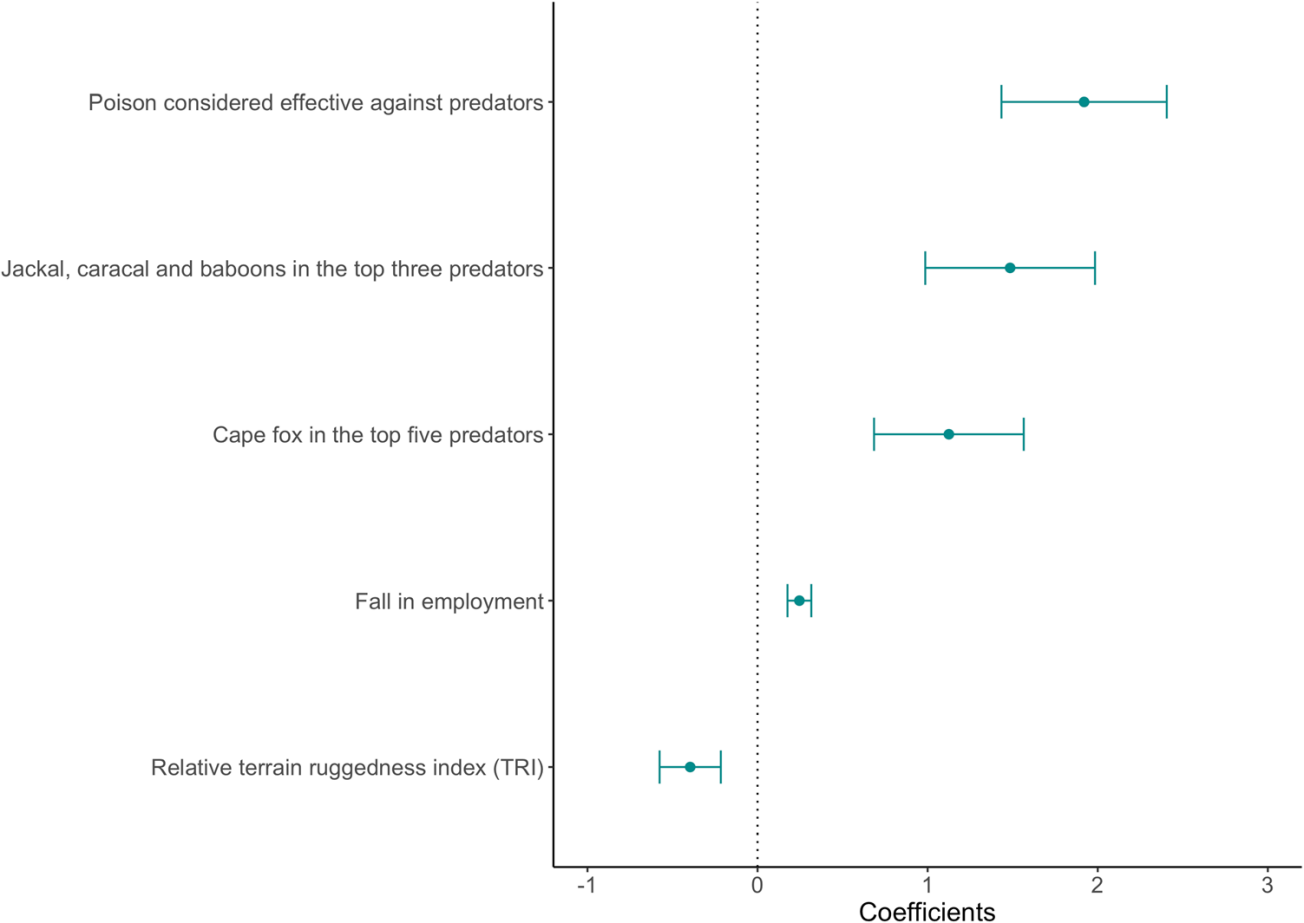
Factors associated with reported poison use by small-livestock farmers in the study area in the South African Karoo. Variable coefficients (dots) and standard errors (lines) are derived from the most parsimonious binomial logistic regression model

After model selection, the most parsimonious binary logistic regression explaining farmers’ reported use of poison contained five statistically significant variables (Table 3, Fig. 6, Appendix S6). Falling on-farm employment; having jackal, caracal and baboons as the top three predators; having Cape fox within the top five predators and considering poison to be effective against predation were all positively related to reported poison use. Relative Terrain Ruggedness Index (TRI) of the main farm was negatively associated with reported poison use.

**Table 3:** Average marginal effects (calculated using robust standard errors—RSE and measured as percent gain) for the most parsimonious binomial logistic regression of reported poison-use by commercial small-livestock farmers (*n* = 76) in the South African Karoo. For factor levels, d*f*/d*x* is the discrete change from the base level

Independent variables	Full model				Most parsimonious model after model selection			
	d*f*/d*x*	RSE	*z*	*p*	d*f*/d*x*	RSE	*z*	*p*
Fall in employment on the respondent’s farm(s)	5.463	0.013	4.264	0.000***	5.531	0.014	4.082	0.000***
Total farms size	− 0.032	0.001	− 0.481	0.630				
Jackal, caracal and baboon are in the top three predators	36.51	0.089	4.108	0.000***	34.22	0.099	3.450	0.000***
Cape fox is in the top five predators	26.06	0.086	3.032	0.002**	23.70	0.082	2.900	0.004**
Farmer considers poison effective against predation	46.67	0.105	4.454	0.000***	48.87	0.101	4.834	0.000***
Farmer believes in the dominant jackal pair narrative	− 12.23	0.103	− 1.187	0.235				
Age	− 0.571	0.003	− 1.648	0.099				
Number of years of education	− 3.344	0.034	− 0.985	0.324				
Percentage of lambs lost	1.386	0.222	0.062	0.950				
Relative Terrain Ruggedness Index (TRI)	− 9.145	0.040	− 2.302	0.021*	− 8.908	0.036	− 2.480	0.013*

*p < 0.050, **p < 0.010, ***p < 0.001 for *z* values

Having jackal, caracal and baboons as the top three predators, and considering poison as an effective method to reduce predation exhibited the strongest marginal effects (34.22 ± 0.10% and 48.87 ± 0.10%, respectively) amongst all categorical variables. Ranking Cape fox in the top five predators on the farm increased the average marginal effect of farmers reporting poison use (23.7 ± 0.08%; Table 3). A decrease of one farm worker on the farm since the farmer had starting farming was associated with a 5% (interquartile range: 0.03–0.08%) increase in the probability of reporting poison use. An increase of one unit in the relative Terrain Ruggedness Index (TRI) was associated with a 9% (interquartile range: 0.02–0.16%) reduction in the probability of reporting poison use (Table 3). None of the socio-economic characteristics, including percentage of lamb losses, were retained in the most parsimonious model (Appendix S6).

## Discussion

We used ethnographic field observations and semi-structured interviews to investigate commercial small-livestock farmers’ reported experience with and perceived effectiveness of lethal versus non-lethal methods for reducing predation on their farms in the Central Karoo. Farmers were willing to discuss the use of control methods, including poison, a result also reported by Santangeli et al. (2016) who used an indirect questioning technique. Farmers had more experience with lethal predator control, which they perceived to be cheaper and more effective than non-lethal methods. Reported poison use was widespread despite being illegal and the highest recorded so far in southern Africa. Poison was easily available, perceived to be effective and its use on extensive farmlands was rarely policed. It was often employed at the first sign of predators on the farm. More than half (53%) reported having used poison and 17% reported using it regularly. That this is consistent with results from a longitudinal economic survey in the region (Nattrass and Conradie 2018) lends confidence to our findings. However, as both surveys used direct questioning methods, these estimates should be regarded as lower bound estimates.

Farmers’ experience and perceived effectiveness of predation control methods.

An international review has revealed that livestock farmers typically prefer lethal over non-lethal management of the risk predators pose to their livestock (Moreira-Arce et al. 2018). This is also true in South Africa (Nattrass and Conradie 2018; Brink et al 2021). Whereas Nattrass and Conradie (2018) found that poison use was positively linked to livestock predation (indicating that it was retaliatory), our study revealed a positive sign but not a statistically significant relationship. This difference may be attributed to the different methods employed by the two surveys to measure livestock losses (Nattrass et al 2020a).

Farmers targeted predators as a species (i.e., jackal, caracal) rather than specific “problem” individuals that may be responsible for losses. More than half the respondents (57%) considered that “the only good carnivore is a dead carnivore”, similar to what Lucherini and Merino (2008) noted in the High Andes of Argentina. This strong preference for eliminating predators was evident in everyday discussions, becoming in effect a social norm (e.g., “If I have “ongediertes” [i.e., negative Afrikaans term to talk about predators] on my farm, then I have livestock losses, so for me a dead jackal is a good jackal. It means that my lambs stay alive” Farmer #68).

Similar to Brink et al. (2021) who found that the strongest predictor of poison-use was whether farmers perceived it as being a common practice among their peers, participant observation highlighted that farmers in our area were using management techniques consistent with their social norms and peer pressure (i.e., all used the same lethal control methods and looked at what their neighbors and friends were using, e.g., “If my neighbor tries a new method and tells me it works, I will try it too. We need to have less losses or we won’t survive, even if it means using poison.” Farmer #69), based on their belief that the method will work (i.e., perceived effectiveness), their past experiences (i.e., they learnt the methods with their fathers and those used to work in the past, Nattrass and Conradie 2015), their confidence in being able to effect the method, and their needs (i.e., to control predator numbers on their own or with their neighbors to limit livestock losses on their farms). Farmers who did not conduct lethal control were frowned upon (as providing “breeding grounds” for jackals), and most non-lethal measures were looked on with suspicion, skepticism and preconceptions about their cost and effectiveness (e.g., “All these methods that you call non-lethal, they only work if you use many of them and if you change them all the time. They only work for a short time. The jackal is too clever. That will cost me too much money to change my methods all the time. And who will do it? All my workers are busy and I can’t afford to employ more people.” Farmer #45). Farmers worried that non-lethal methods simply deflected predators onto other farms and that if all were to adopt them, it would result in unsustainable additional costs in terms of labor and fencing. In the absence of appropriately scaled, before and after (with control) studies, the relative effectiveness of different methods remains a largely subjective matter fomenting considerable conflict between different stakeholders in the Karoo and globally (Treves et al. 2016; van Eeden et al. 2018).

Herding and livestock guarding dogs (Fig. 1), amongst the most commonly used methods globally (Moreira-Arce et al. 2018), had been trialed by less than 20% of the Central Karoo farmers and scored poorly on perceived effectiveness. Farmers stated it was difficult to find reliable farm workers/herders who would be prepared to stay in the field for extended periods of time to guard livestock. The statutory minimum wage of ZAR 105/day (approximately US$ 7 at the time of the study) for a farm worker who could work as a shepherd was also considered unsustainable. Almost a fifth of the farmers interviewed (*n* = 15) reported now having to use family members for controlling predators, a role historically (prior to the 1990s) fulfilled by farm workers. As for livestock guarding dogs, farmers reported that the large size of the sheep camps, the rather rugged terrain in many areas, and the lack of flocking behavior of the Dorper sheep made it difficult for a dog to provide protection.

### Correlates of reported poison use

Research in South Africa and Kenya has revealed that poison use was a consequence of, as well as a response to, high perceived levels of predation and general concerns about predators posing threats to livestock (Nattrass and Conradie 2018; Brink et al. 2021; Didarali et al. 2022). In our model, reported poison use was best explained by farmers perceived presence and threats of jackal, caracal, baboons and Cape fox on their farms, perceptions of effectiveness of poison in combatting predation and declining employment. Poison was considered by respondents to not only be effective, but also cheap, quick, readily available, easy to use against predators and covert in its action. Similar perceptions have been shown to influence the use of poison by Masaai pastoralists in southern Kenya (Didarali et al. 2022).

That poison use was negatively associated with TRI seems somewhat counter-intuitive as jackals on Karoo farmlands have been shown to prefer rugged and mountainous areas (Drouilly et al. 2018a; Woodgate et al. 2023), which was not the case on various protected areas in the Karoo, where jackals favor flat plains and lower elevations. In addition, livestock predation has been shown to be positively associated with TRI (Nattrass et al 2020a). Together, these results point towards a possible use by jackals of those rugged areas as refuges from human persecution on Karoo farmland. The negative relationship between TRI and reported poison use probably reflects the fact that road access is often limited in rugged mountainous parts of the farm, and hence that farmers will have difficulties identifying and reaching sites on such landscapes frequented by predators.

Nattrass and Conradie (2018) suggested that the use of poison might be a response to increasing economic hardship and restrictive regulations surrounding the management of predators. We agree that economic context matters but are not convinced that regulation of predator control plays a role. There is no oversight regarding what methods farmers use on private farmland and enforcement of the law against illegal methods including poison use is rare. It is also possible that lack of institutional trust (i.e., both towards wildlife authorities and conservation agencies) and reduced government support to farmers has contributed to farmers taking matters into their own hands and resorting to poison (Brink et al. 2021; Viollaz et al. 2021). Many farmers had to reduce staff numbers due to lower farm profitability, declining government support for agriculture and higher operating costs (Conradie et al. 2013). This almost certainly contributed to the abandonment of herding (Nattrass and Conradie 2018), the reduced maintenance of predator-proof fences and to the preference of poison over traps that require at least one worker with adequate training, jackals being particularly hard to catch on farmland (Botha et al. 2022). Under these conditions, poison becomes a convenient, low-cost alternative, especially where farmers perceive it to be effective.

### Resistance to non-lethal methods and potential approaches to mitigate poison use

Farmers in our study preferred using lethal methods to control predation. Other studies have found that, when properly applied, non-lethal methods can be more cost-effective (Treves et al. 2016; Stone et al. 2017). Understanding how farmers make decisions about which methods to use is essential to shifting behavior and for the uptake of novel approaches (Vogel et al. 2022). It has been suggested that for farmers to transition to non-lethal control, they will firstly need real-world demonstrations of the effectiveness of non-lethal methods (Moreira-Arce et al. 2018). However, if non-lethal methods entail hiring more workers, farmers in the Karoo will be resistant because this would require further elevation of input costs when they are already experiencing a cost-price squeeze that has reduced overall productivity (Conradie et al. 2013). Labor carries the additional risk of individual claiming rights to permanently reside on the land (DLA 1997).

Like other illicit behaviors, illegal poisoning of carnivores is multifaceted and complex to combat due to its cryptic nature. In southern Kenya, the lower prevalence of poison use amongst pastoralists aware of the Wildlife Act (Didarali et al. 2022) suggests that legislation can serve as a deterrent from using poison, even when legislation is poorly enforced. Behavioral change campaigns where farmers are reminded that poison use is a punishable criminal offense, where collateral victims are highlighted—as we recorded that at least some farmers are sensitive to this issue—and where the aesthetic appeal of predators is promoted (Drouilly et al. 2021) could have a role to play in curbing poison use. Providing farmers with evidence of the effectiveness of alternative livestock loss prevention methods will also be key (van Eeden et al. 2018). Working with trusted messengers, such as agricultural colleges and farmers associations, and using a participatory approach to transform the main stakeholders into active elements of the fight against the use of poison could be beneficial to ensure protection of existing biodiversity on farmland and to direct the needed shift from current practices to more pro-biodiversity management practices.

## Conclusion

Despite being an illegal practice in many countries, poison remains amongst the most readily available predation control methods for farmers. Farmers, as land managers, have a critical role to play for biodiversity conservation and ecosystem functioning outside of protected areas. Karoo farmers currently coexist with a diversity of wild animals on their farms (Drouilly et al. 2018a; Woodgate et al. 2018), but for them to become more meaningful custodians of biodiversity, outside encouragement and assistance will be required to enable them to trial alternative, less harmful methods. When addressing the threat represented by illegal poison use, it is important to understand the social and economic context as well as the attitudes that underpin it, in particular the limitations and reservations that currently prevent farmers from adopting these methods. Shifting away from poison use and other lethal practices is likely to require interventions demonstrating the effectiveness of alternatives, and perhaps also financial assistance.

## Supplementary Information

Below is the link to the electronic supplementary material.

Supplementary file1 (PDF 325 KB)
