# Supplementary material for: Small-livestock farmers’ perceived effectiveness of predation control methods and the correlates of reported illegal poison use in the South African Karoo
Source: Ambio. 2023 Jun 30;52(10):1635–49. doi: 10.1007/s13280-023-01892-7 (PMC10460750; doi:10.1007/s13280-023-01892-7)
Supplement: Supplementary file 1 — Supplementary file1 (PDF 325 KB) [file 13280_2023_1892_MOESM1_ESM.pdf]

AMBIO

Electronic Supplementary Material
This supplementary material has not been peer reviewed.

Title: **Small-livestock farmers' perceived effectiveness of predation control methods and the correlates of reported illegal poison use in the South African Karoo**

Authors: Marine Drouilly, Nicoli Nattrass M. Justin O'Riain

Appendix S1: Ethical considerations before and after the interview process

Although most farmers were familiar with our research, we re-discussed the project with them before each interview and talked them through the potential risks of participating (i.e., notably discussing their use of illegal control methods with us). Participants were made aware of their right to participate voluntarily or to decline. We explained that responses to all questions would remain confidential by anonymizing data after collection and removing any other unnecessary personal details from interview transcripts (Knott et al. 2022). We informed them that results would be presented as percentages. Free, informed (including the intended uses of the research) oral consent, was obtained from every participant, both at the time of making the appointment and just before the interview itself (Puri 2010). We explained to participants that they could withdraw their consent at any time during the interview and for a week afterwards. We also indicated that anything they would like to tell us could be done off the record and/or not disseminated. Finally, we made sure that participants would not feel judged when they shared information with us, notably on illegal practices (Knott et al. 2022).

Appendix S2: Validation of species identification and definition of important terms used in the interviews

Before asking questions about wildlife, we ensured that farmers could correctly identify the species of interest, by showing them pictures of the different species and asking them to identify the species. All farmers could correctly identify black-backed jackal (*Canis mesomelas*), Cape fox (*Vulpes chama*), bat-eared fox (*Otocyon megalotis*), aardwolf (*Proteles cristatus*), caracal (*Caracal caracal*), African wildcat (*Felis lybica*), chacma baboons (*Papio ursinus*), aardvark (*Orycteropus afer*) and Cape porcupine (*Hystrix africaeaustralis*). Other groups of species, such as mongooses, eagles, etc. could not always be accurately identified at the species level and were therefore grouped together in our interviews. Similarly, we ensured that the terms that we used for all the control methods (e.g., conibear trap, poisoned collar) were defined and/or photographs of the methods shown to the farmers before recording their answers, to make sure that there was no ambiguity.

Appendix S3: Interview guide with the set of questions relevant to the paper

We used a questionnaire that combined a pre-determined set of close-ended and open-ended questions as a guide for our interviews with farmers. The latter prompted discussion, with the opportunity for the interviewer to explore particular themes of responses further. Although the questions about poison were all close-ended, the interviewer exercised "opportunistic discretion while asking questions in order to be flexible and sensitive to the flow of the conversation and each farmer's particularities" (*sensu* Nair et al. 2021). Farmers often discussed particular aspects of using poison further, and the interviewer encouraged them to do so by asking additional questions that could vary from one respondent to the other, allowing the respondent time to open up about this sensitive issue.
Note: all questions were not necessarily asked to all interviewees and the exact order of the questions might have varied from one interview to the other to respect the flow of the conversation.

1) Date of the interview
2) Farm name *(ask for the names of all the farms of the respondents and ask them to place them on the map)*
3) Interviewee's name
4) Interviewee's gender
5) Interviewee's date of birth *(ask year of birth or age if preferred by farmers)*
6) Phone number/email
7) Is sheep farming your main source of income? *(Ask about other land tenures/activities if needed)*
8) How many years of farming experience do you have? In the Karoo?
9) What is your highest level of formal education? *(ask about name of college/institute to see if the respondents went to the same place for their formal education)*
10) What is your religion, if any?
11) How many people were/are employed permanently on your land at the following three points in time?
    a) When you started farming?
    b) In the early 1990s?
    c) At the time of this interview?
12) Did these permanent employees live on your land in the early 1990s?

If there has been a substantial reduction in fulltime employment since the early 1990s, ask the following:

13) What do you attribute the reduction in fulltime employment since the early 1990s to?
14) Do you currently use family labour for tasks that previously would have been done by regular staff ? If the response to the question above is positive, ask the respondents to give examples of the tasks that are now done by family members that used to be done by workers *(look out for tasks related to predators)*
15) Do you have livestock/domestic animal losses on your farm? *(ask about the year prior to the interview)*
16) According to you, what is the biggest cause of livestock losses on your farm?
17) When was the first time you saw a jackal or jackal spoor on your land?
18) Is jackal a problem on your farm? Why? *(look out for information on predation)*
19) When did jackal become a problem for lamb loss on your farm? *(ask only if farmer says so before)*
20) When did lamb loss to jackal become a serious concern to the viability of your farm (i.e., a serious problem)? *(ask only if farmer says so before)*
21) When was the first time you saw a caracal or caracal spoor on your land?
22) Is caracal a problem on your farm? Why? *(look out for information on predation)*
23) When did caracal become a problem for lamb loss on your farm? *(ask only if farmer says so before)*
24) When did lamb loss to caracal become a serious concern to the viability of your farm (i.e., a serious problem)? *(ask only if farmer says so before)*
25) When was the first time you saw a baboon or baboon spoor on your land?
26) Are baboons a problem on your farm? Why? *(look out for information on predation)*

27) When did baboon become a problem for lamb loss on your farm? *(ask only if farmer says so before)*
28) When did lamb loss to baboons become a serious concern to the viability of your farm (i.e., a serious problem)? *(ask only if farmer says so before)*
29) Do you think jackals have increased in the Central Karoo over the last decade? If yes, what could be the reasons, according to you?
30) Do you think caracals have increased in the Central Karoo over the last decade? If yes, what could be the reasons, according to you?
31) Do you think baboons have increased in the Central Karoo over the last decade? If yes, what could be the reasons, according to you?
32) What evidence do you use to infer culpability of a particular predator species?
33) How many livestock heads/ewes do you have?
34) According to you, what animal causes the most predation damage on your farm? *(show respondents pictures of these species and check that they can identify them properly)*
    a) Jackal
    b) Caracal
    c) Leopard
    d) Baboon
    e) Cape fox
    f) African wildcat
    g) Other *(ask them to specify)*
35) According to you, what animal causes the most infrastructure damage on your farm? *(show respondents pictures of these species and check that they can identify them properly)*
    a) Porcupine
    b) Aardvark
    c) Aardwolf
    d) Baboon
    e) Other *(ask them to specify)*
36) Please, rank the following animals from 1 to 6 with 1 being the animal that causes the most predation damage on your farm and 6 being the animal that causes the less damage *(Do not rank animals that don't cause damage on your farm):*
    Jackal Caracal Leopard Baboon Cape fox African wildcat Other *(specify)*
37) Do you attempt to limit livestock predation on your farm?
38) If yes, please, indicate the effectiveness of each of the following predator control methods for the control of <u>caracal predation on your farm</u> *(Effectiveness of a method is understood as simultaneously being effective in killing predators and protecting your sheep, as was discussed during the farmer meeting in May 2014)*

1= method does not work for me
5 = method works very well for me
0 = no experience of this method

a. Gin trap	1	2	3	4	5	0
b. Cage	1	2	3	4	5	0
c. Conibear trap *(Doodslaner; show picture)*	1	2	3	4	5	0
d. Poison 1080	1	2	3	4	5	0
e. Poison aldicarb (two-step)	1	2	3	4	5	0
f. Other poison	1	2	3	4	5	0
g. Poisoned collar	1	2	3	4	5	0
h. Cyanide device/Coyote getter *(show picture)*	1	2	3	4	5	0
i. Call and shoot at night	1	2	3	4	5	0
j. Helicopter hunting	1	2	3	4	5	0
k. Day hunting	1	2	3	4	5	0
l. Hunting with dogs	1	2	3	4	5	0
m. Bells on collar *(show picture)*	1	2	3	4	5	0
n. Ribbons on collar *(show picture)*	1	2	3	4	5	0
o. Protective collars *(show picture)*	1	2	3	4	5	0
p. Olfactive repellent	1	2	3	4	5	0
q. Docking tail for lambs but not in the field	1	2	3	4	5	0
r. Disposing of livestock carcasses	1	2	3	4	5	0
s. Shorter lambing season	1	2	3	4	5	0
t. Single lambing season	1	2	3	4	5	0
u. Coordinated lambing season with neighbors	1	2	3	4	5	0
v. Herder	1	2	3	4	5	0
w. Livestock guarding dog	1	2	3	4	5	0
x. Livestock guarding donkey	1	2	3	4	5	0
y. Livestock guarding ostriches	1	2	3	4	5	0
z. Acoustic repellent device	1	2	3	4	5	0
aa. Light repellent device	1	2	3	4	5	0
bb. Acoustic and light repellent device	1	2	3	4	5	0
cc. Increase small mammal availability	1	2	3	4	5	0
dd. Keep resident caracal	1	2	3	4	5	0
ee. Keep resident leopard	1	2	3	4	5	0

39) If yes, please, indicate the effectiveness of each of the following predator control methods for the control of jackal predation on your farm *(Effectiveness of a method is understood as simultaneously being effective in killing predators and protecting your sheep, as was discussed during the farmer meeting in May 2014)*

1= method does not work for me
5 = method works very well for me
0 = no experience of this method

a. Gin trap	1	2	3	4	5	0
b. Cage	1	2	3	4	5	0
c. Conibear trap *(Doodslaner; show picture)*	1	2	3	4	5	0
d. Poison 1080	1	2	3	4	5	0
e. Poison aldicarb (two-step)	1	2	3	4	5	0
f. Other poison	1	2	3	4	5	0
g. Poisoned collar	1	2	3	4	5	0

h. Cyanide device/Coyote getter *(show picture)*	1	2	3	4	5	0
i. Call and shoot at night	1	2	3	4	5	0
j. Helicopter hunting	1	2	3	4	5	0
k. Day hunting	1	2	3	4	5	0
l. Hunting with dogs	1	2	3	4	5	0
m. Bells on collar *(show picture)*	1	2	3	4	5	0
n. Ribbons on collar *(show picture)*	1	2	3	4	5	0
o. Protective collars *(show picture)*	1	2	3	4	5	0
p. Olfactive repellent	1	2	3	4	5	0
q. Docking tail for lambs but not in the field	1	2	3	4	5	0
r. Disposing of livestock carcasses	1	2	3	4	5	0
s. Shorter lambing season	1	2	3	4	5	0
t. Single lambing season	1	2	3	4	5	0
u. Coordinated lambing season with neighbors	1	2	3	4	5	0
v. Herder	1	2	3	4	5	0
w. Livestock guarding dog	1	2	3	4	5	0
x. Livestock guarding donkey	1	2	3	4	5	0
y. Livestock guarding ostriches	1	2	3	4	5	0
z. Acoustic repellent device	1	2	3	4	5	0
aa. Light repellent device	1	2	3	4	5	0
bb. Acoustic and light repellent device	1	2	3	4	5	0
cc. Increase small mammal availability	1	2	3	4	5	0
dd. Keep resident jackal	1	2	3	4	5	0
ee. Keep resident leopard	1	2	3	4	5	0

40) Do you think poison is effective against predation? Yes No Unsure

41) Have you used poison on your farm in the last five years?

a. Yes, regularly *(i.e., at least once a month)*

b. Yes, as last resort *(i.e., when no other methods such as calling and shooting at night has been effective in limiting livestock losses)*

c. Not using poison

d. Never

e. Prefer not to answer

42) How do you usually respond to <u>fresh signs</u> of predators?

Mark all that apply	*Jackal*	*Caracal*
a. Do nothing; leave them alone	____________	______
b. Wait for damage before doing anything	____________	______
c. Immediately deploy additional trackers	____________	______
d. Immediately deploy a professional hunter/trapper	____________	______
e. Immediately set off on a hunt yourself/set traps	____________	______
f. Immediately remove livestock from danger zone	____________	______
g. Immediately send out a herder	____________	______
h. Immediately deploy poison	____________	______
i. Unsure	____________	______
j. Other *(specify)*	____________	______

Discuss the "dominant pair" theory with the interviewees

43) Do you think that killing the dominant/territorial pair of jackals on your farm will increase the number of jackals on your farm *(and hence the losses on your farms)*?
44) Do you think that non-lethal methods to protect sheep from predators are more or less expensive than lethal methods *(i.e., killing predators)*?
45) What is the first thing you think about when I say "jackal"?_____________
46) What is the first thing you think about when I say "caracal"?____________
47) Do you think that if you remove/kill a predator, another one will take its place? *(related to question # 43), but not specific to jackals – discuss compensatory life-history traits with respondent)*

Appendix S4: Statistical analysis – Model selection of the correlates of reported poison use

To avoid collinearity among the continuous variables in the binary logistic regression, we calculated Spearman's correlation coefficients (r) for pairs of variables and only included variables where $r < 0.7$ (Dormann et al. 2013). Remaining variables were included in a multivariate global model from which we generated and ranked models with all combinations of predictor variables based on Akaike's Information Criterion, which was adjusted for small sample size (AICc) (Akaike 1974; Burnham and Anderson 2002), using the "MuMIN" package (Barton 2013), and also computed the Bayesian Information Criterion (BIC) (Aho et al. 2014). We checked the top model for multicollinearity among variables by assessing Variance Inflation Factors (VIF) using the "car" package (Fox and Weisberg 2011) and checked that the VIF < 2. Finally, we tested for model goodness-of-fit using a combination of overall goodness-of-fit (using the Hosmer-Lemeshow test; $p < 0.05$), Pseudo-R^2 estimate (Hu et al. 2006) and log likelihood ratio ($p < 0.05$). For each predictor variable, we calculated the average marginal effect (AME; more informative and intuitive than odds-ratios; Norton and Dowd 2018) in the "margins" package (Leeper 2017; Leeper et al. 2018) with robust standard errors (Wooldridge (2010) using heteroscedasticity and autocorrelation consistent estimators implemented in the package "sandwich" (Zeileis 2004; Zeileis 2006). The AME shows the change in probability when the predictor variable increases by one unit. For continuous variables, this represents the instantaneous change given that the "unit" may be very small. For binary variables, the change is from 0 to 1. Each AME is interpreted as percentage points.

The variables selected for multivariate GLMs did not show evidence of collinearity ($r \leq 0.7$ for pairs of continuous variables). All predictor variables under the most parsimonious model exhibited generalized VIF values < 2.0.

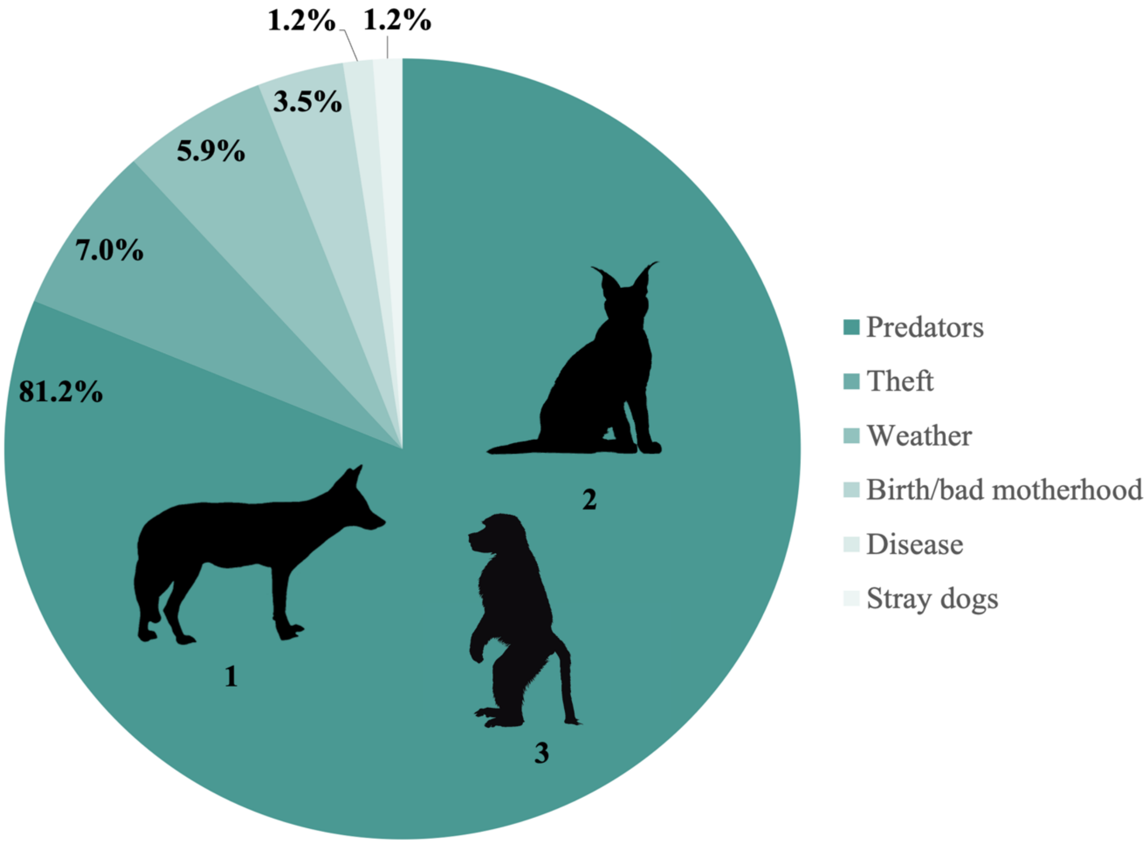

Appendix S5: Diagram representing the percentage of answers when asking the farmers (n=77) what the main cause of livestock losses was on their farms in the Karoo. The silhouettes of the three predators perceived by farmers in our study area to be responsible for the most livestock losses are shown (i.e., black-backed jackal, followed by caracal, and chacma baboon).

Appendix S6: Results of the binomial logistic regression analysis of reported poison-use for predator control by interviewed commercial small-livestock farmers in the South African Karoo in 2014. Model coefficients, standard error and p-values (Pr(>|z|)) are displayed.

	Full model				Most parsimonious model after model selection			
Independent variables	Estimates	Std. Error	z value	Pr(>|z|)	Estimates	Std. Error	z value	Pr(>|z|)
Intercept	2.279	2.631	0.866	0.386	-1.394	0.516	-2.700	0.007 **
Fall in employment on the respondent's farm(s)	0.254	0.068	3.750	0.000 ***	0.246	0.070	3.532	0.000 ***
Total farms size	-0.001	0.003	-0.479	0.632				
Jackal, caracal and baboon are in the top three predators	1.658	0.511	3.246	0.001 **	1.485	0.499	2.975	0.003 **
Cape fox is in the top five predators	1.327	0.542	2.449	0.014 *	1.125	0.440	2.554	0.011 *
Farmer considers poison efficient against predation	1.943	0.59	3.260	0.001 **	1.920	0.486	3.951	0.000 ***
Farmer believes in the dominant jackal pair narrative	-0.552	0.471	-1.173	0.241				
Age	-0.027	0.017	-1.540	0.124				
Number of years of education	-0.155	0.160	-0.971	0.331				
Percentage of lambs lost	0.064	1.034	0.062	0.950				
Relative Terrain Ruggedness Index (TRI)	-0.425	0.212	-2.001	0.045 *	-0.396	0.180	-2.203	0.028 *
Number of observations	66				69			
Log likelihood	-25.542				-27.95			
Prob > Chi^2	<0.001				<0.001			
AIC_c	77.97				69.26			
BIC	97.17				81.31			
Nagelkerke's R^2	0.61				0.58			

References:

Aho, K., D. Derryberry, and T. Peterson. 2014. Model selection for ecologists: The worldviews of AIC and BIC. *Ecology* 95: 631–636.

Akaike, H. 1974. A new look at the statistical model identification. *IEEE Transactions on Automatic Control* 19: 716–723.

Barton, K. 2013. *Package "MuMIn": Multi-modal inference. Model selection and model averaging based on information criteria (AICc and alike). R package version 1.42.1. Reference manual.*

Burnham, K., and D. Anderson. 2002. *Model selection and multimodel inference: A practical information-Theoretic approach*. 2nd ed. New-York: Springer-Verlag.

Dormann, C. F., J. Elith, S. Bacher, C. Buchmann, G. Carl, G. Carré, J. R. G. Marquéz, B. Gruber, et al. 2013. Collinearity: A review of methods to deal with it and a simulation study evaluating their performance. *Ecography* 36: 27–46.

Fox, J., and S. Weisberg. 2011. *Multivariate linear models in R. An R Companion to Applied Regression.* Los Angeles: SAGE publications, Inc.

Hu, B., J. Shao, and M. Palta. 2006. Pseudo-R^2 In Logisitc Regression Model. *Statistica Sinica* 16: 847–860.

Knott, E., A. Rao, K. Summers, and C. Teeger. 2022. Interviews in the social sciences. *Nature Reviews Methods Primers* 2: 1–15.

Leeper, T., J. Arnold, and V. Arel-Bundock. 2018. *Package "margins": Marginal Effects for Model Objects. R package version 0.3.23. Reference manual.*

Leeper, T. J. 2017. *Interpreting regression results using average marginal effects with R's margins. Reference manual.*

Nair, R., Patil, O., Surve, N., Andheria, A., Linnell, J. D., & Athreya, V. (2021). Sharing spaces and entanglements with big cats: The Warli and their Waghoba in Maharashtra, India. Frontiers in Conservation Science, 21.

Norton, E. C., and B. E. Dowd. 2018. Log Odds and the Interpretation of Logit Models. *Health Services Research* 53: 859–878.

Puri, R. 2010. Participant observation. In *Conducting research in conservation*, ed. H. Newing, 107–119. Oxon, UK: Routledge.

Wooldridge, J. 2010. *Econometric Analysis of Cross Section and Panel Data*. 2nd ed. Cambridge, USA: MIT Press.

Zeileis, A. 2004. Econometric computing with HC and HAC covariance matrix Estimators. *Journal of Statistical Software* 11: 1–17.

Zeileis, A. 2006. Object-Oriented Computation of Sandwich Estimators. *Journal of Statistical Software* 16: 1–16.
